# Standardizing *Plasmodium falciparum* infection prevalence measured via microscopy versus rapid diagnostic test

**DOI:** 10.1186/s12936-015-0984-9

**Published:** 2015-11-17

**Authors:** Bonnie Mappin, Ewan Cameron, Ursula Dalrymple, Daniel J. Weiss, Donal Bisanzio, Samir Bhatt, Peter W. Gething

**Affiliations:** Spatial Ecology and Epidemiology Group, Department of Zoology, University of Oxford, Tinbergen Building, Oxford, OX1 3PS UK

**Keywords:** Malaria, *Plasmodium falciparum*, Africa, RDT, Microscopy, Diagnostics

## Abstract

**Background:**

Large-scale mapping of *Plasmodium falciparum* infection prevalence relies on opportunistic assemblies of infection prevalence data arising from thousands of *P. falciparum* parasite rate (*Pf*PR) surveys conducted worldwide. Variance in these data is driven by both signal, the true underlying pattern of infection prevalence, and a range of factors contributing to ‘noise’, including sampling error, differing age ranges of subjects and differing parasite detection methods. Whilst the former two noise components have been addressed in previous studies, the effect of different diagnostic methods used to determine *Pf*PR in different studies has not. In particular, the majority of *Pf*PR data are based on positivity rates determined by either microscopy or rapid diagnostic test (RDT), yet these approaches are not equivalent; therefore a method is needed for standardizing RDT and microscopy-based prevalence estimates prior to use in mapping.

**Methods:**

Twenty-five recent Demographic and Health surveys (DHS) datasets from sub-Saharan Africa provide child diagnostic test results derived using both RDT and microscopy for each individual. These prevalence estimates were aggregated across level one administrative zones and a Bayesian probit regression model fit to the microscopy- versus RDT-derived prevalence relationship. An errors-in-variables approach was employed to account for sampling error in both the dependent and independent variables. In addition to the diagnostic outcome, RDT type, fever status and recent anti-malarial treatment were extracted from the datasets in order to analyse their effect on observed malaria prevalence.

**Results:**

A strong non-linear relationship between the microscopy and RDT-derived prevalence was found. The results of regressions stratified by the additional diagnostic variables (RDT type, fever status and recent anti-malarial treatment) indicate that there is a distinct and consistent difference in the relationship when the data are stratified by febrile status and RDT brand.

**Conclusions:**

The relationships defined in this research can be applied to RDT-derived *Pf*PR data to effectively convert them to an estimate of the parasite prevalence expected using microscopy (or vice versa), thereby standardizing the dataset and improving the signal-to-noise ratio. Additionally, the results provide insight on the importance of RDT brands, febrile status and recent anti-malarial treatment for explaining inconsistencies between observed prevalence derived from different diagnostics.

## Background

Large-scale maps of *Plasmodium falciparum* infection prevalence [1–3] are increasingly used to inform disease control planning, implementation and evaluation at national to global scales [4], and as a basis for disease burden estimation and the monitoring of progress towards international targets [4–7]. Mapping at the continental or global scale relies on opportunistic assemblies of data on infection prevalence arising from thousands of *P. falciparum* parasite rate (*Pf*PR) surveys conducted in different countries. The between-site variance observed in these *Pf*PR estimates arises from both an underlying signal component: the variation of the true infection prevalence and the residual noise component, attributable, not only to the inherent random error, but also to a range of confounding factors that reduce the comparability of *Pf*PR measurements from different surveys, most notably, immunological differences in the age ranges of subjects surveyed [8] and differences in parasite detection methods. Whilst random error and age-standardization have been addressed in previous mapping efforts, the effect of different diagnostic methods has not. Previous research has defined the functional relationship between microscopy and polymerase chain reaction (PCR) detection [9], but little research has explored the functional differences between microscopy and rapid diagnostic test (RDT). Given that the majority of *Pf*PR data are based on positivity rates measured by either microscopy or RDT, and that the sensitivity and specificity of these approaches are not identical [10], it is crucial to understand the functional differences between these two approaches.

**Fig. 1:**
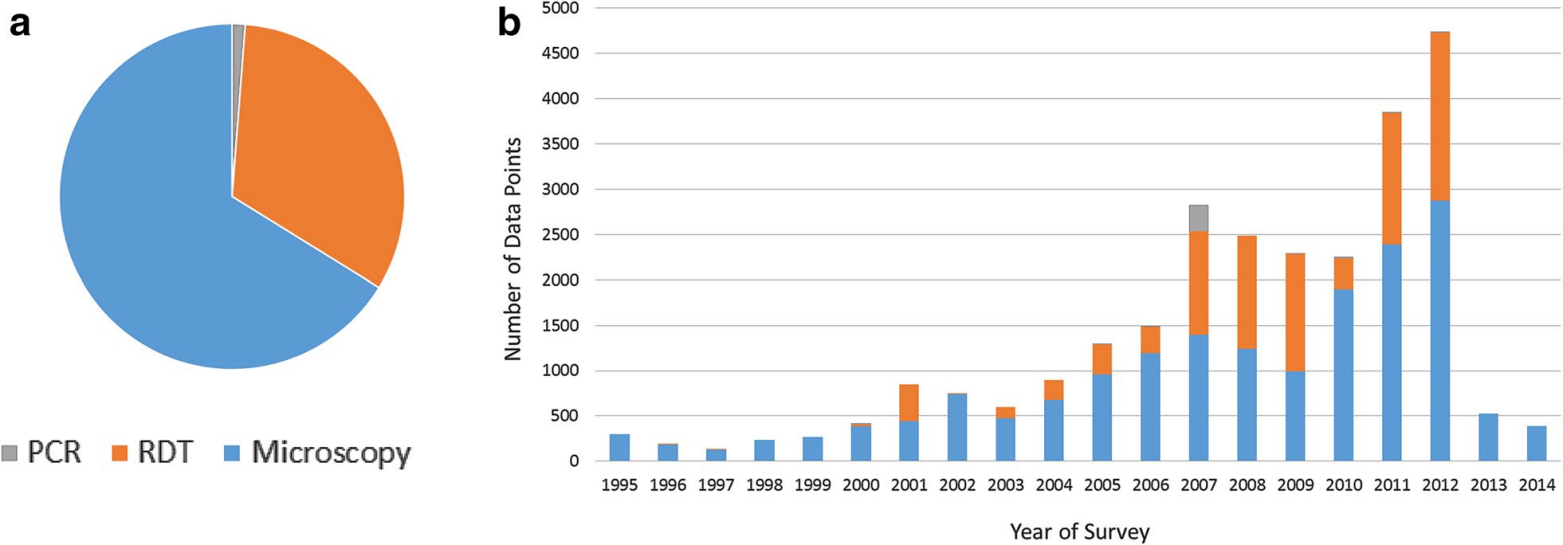
Breakdown of African *Pf*PR data points in the MAP database by diagnostic type [polymerase chain reaction (*PCR*), rapid diagnostic test (*RDT*) and microscopy] as of November 2014. **a** Overall proportion, and **b** a time series by survey year from 1995 to 2014. The reduced numbers in 2013 and 2014 are to be expected due to the lag time between data collection and its subsequent release

Detection of parasite infection via microscopy has formed the mainstay of modern *Pf*PR surveys for many decades [11]. Giemsa-stained thick smear microscopy under ideal conditions where stains are prepared correctly and the slide is analysed by an expert is highly accurate for infection diagnosis at densities above 100 parasites per µL [12]. Since the early 2000s, however, economically preferable RDTs have become widely used in field locations as they allow quick diagnosis through antigen-detection without the need for laboratory equipment. Figure 1 illustrates the increasing role of RDT measurements in the global assembly of *Pf*PR surveys maintained by the Malaria Atlas Project (MAP). This increase is mirrored in clinical settings, where the introduction of RDTs has enabled improvements in the proportion of suspected malaria cases receiving parasitological diagnosis (e.g., rising from 20 to 62 % between 2006 and 2013 in the African public sector and with over half of reported cases in 2013 identified by RDTs [4]).

World Health Organization (WHO) guidelines state that all RDTs used for malaria diagnosis pass the following criteria in the WHO Malaria RDT product testing programme: have a panel detection score of at least 75 % at 200 parasites per µL, with a false positive rate <10 and <5 % of tests deemed invalid [13]. RDTs used in *Pf*PR surveys undergo extensive testing as part of WHO quality assurance procedures [14]. However, despite these procedures, the relative performance of RDTs *versus* microscopy in the field is influenced by a wide range of factors that cannot be fully captured in standardized quality assurance tests. RDTs have been shown to become ineffective through poor storage [15], and can display false positivity due to the perseverance of antigens detected in the blood after both anti-malarial treatment and parasite clearance [16, 17]. Despite these limitations, no simple adjustment factors currently exist to functionally map between RDT-derived *Pf*PR measurements and those likely to be observed using microscopy.

To address the need for cross-comparability of microscopy- and RDT-derived *Pf*PR measurements, a large database of individual-level parasitological outcomes tested, using both methods, was assembled from national household surveys conducted in sub-Saharan Africa. A hierarchical Bayesian model was then developed to capture the functional divergence in *Pf*PR measured using the two techniques. The extent of this divergence was further explored in sub-analyses stratified using the presence or absence of symptomatic infection (i.e., fever), recent treatment with effective anti-malarial drugs and the type and brand of RDT used.

## Methods

### Data collection

The Demographic and Health Surveys (DHS) Programme collects nationally representative health and socio-economic information from over 90 countries worldwide through cross-sectional household surveys [18]. Typically, malaria testing within DHS Programme is limited to children under five years old. For this study, children from sub-Saharan African DHS datasets, whose malaria diagnostic outcome was recorded using both microscopy and RDT, were selected. Table 1 lists the 25 DHS Programme that met these inclusion criteria as of 15 July, 2015, which were tabulated from 118,078 individuals tested for falciparum malaria. The administrative zone (ADMIN1) of residence and the RDT and microscopy diagnostic outcome was recorded for each individual. Where available, the following additional factors were extracted: (1) RDT brand; (2) RDT type used whether the RDT detected histidine-rich protein 2 (HRP2) or HRP2 and pan-*Plasmodium* lactate dehydrogenase (pLDH) or HRP2 and *Plasmodium vivax*-specific pLDH; (3) whether the individual had been febrile within the last two weeks; and (4) whether febrile individuals had received treatment with artemisinin-based combination therapy (ACT) anti-malarial within the previous 2 weeks.

**Table 1:** Surveys included in analysis, the number of individuals included within each survey, the RDT type, the RDT brand, the RDT model if known, and the age range of patients screened for parasitaemia within each survey

Country	Survey year	Type of survey	No of individuals	No of ADMIN1	RDT type	RDT brand	RDT model	Age range of individuals
Angola	2011	MIS	3322	21	HRP2/Pv-pLDH	SD Bioline	05FK80	6–59 months
Benin	2011–2012	Standard DHS	3736	12	HRP2	Paracheck	30301100	1–59 months
Burkina Faso	2010	Standard DHS	5741	13	HRP2	Paracheck	30301100	6–59 months
Burundi	2012	MIS	3718	18	HRP2/pan-pLDH	SD Bioline	05FK60	6–59 months
Cote d’Ivoire	2011–2012	Standard DHS	3927	14	HRP2	SD Bioline	05FK50	6–74 months
Democratic Republic of Congo	2013–2014	Standard DHS	8135	26	HRP2	SD Bioline	05FK50	6–59 months
The Gambia	2013	Standard DHS	3262	8	HRP2/pan-pLDH	SD Bioline	Unknown	6–59 months
Guinea	2012	Standard DHS	3198	8	HRP2/pan-pLDH	First Response	I16FRC30	6–59 months
Liberia	2009	MIS	4960	18	HRP2	Paracheck	Unknown	6 months–6 years
Liberia	2011	MIS	3060	18	HRP2	First Response	I13FRC30	6–59 months
Madagascar	2011	MIS	6821	23	HRP2/pan-pLDH	CareStart	Unknown	6–64 months
Madagascar	2013	MIS	6147	22	HRP2/pan-pLDH	CareStart	Unknown	6–65 months
Malawi	2012	MIS	2105	3	HRP2	SD Bioline	05FK50	6–59 months
Mali	2010	Special EA&P	1716	9	HRP2	Paracheck	Unknown	6–59 months
Mali	2012–2013	Standard DHS	5640	6	HRP2	Paracheck	30301100	0–83 months
Mozambique	2011	Standard DHS	4891	13	HRP2/Pv-pLDH	SD Bioline	05FK80	6–59 months
Nigeria	2010	MIS	5127	38	HRP2	Paracheck	30301100	6–59 months
Rwanda	2010	Standard DHS	4887	5	HRP2	First Response	I13FRC30	6–73 months
Senegal	2008–2009	MIS	3947	15	HRP2	Paracheck	30301100	6–59 months
Senegal	2010–2011	Standard DHS	4603	14	HRP2	Paracheck	30301100	6–77 months
Senegal	2012–2013	Continuous DHS	7252	14	HRP2/pan-pLDH	SD Bioline	05FK60	6–76 months
Senegal	2014	Continuous DHS	6760	14	HRP2/pan-pLDH	SD Bioline	Unknown	6–59 months
Tanzania	2011–2012	Standard AIS	7264	30	HRP2/pan-pLDH	SD Bioline	05FK60	6–59 months
Togo	2013–2014	Standard DHS	3861	6	HRP2/pan-pLDH	First Response	Unknown	6–59 months
Uganda	2009	MIS	3998	95	HRP2	Paracheck	30301100	0 month–5 years

*MIS* Malaria Indicator Survey, *DHS* Demographic Health Survey, *EA&P* anaemia and malaria parasitaemia survey

### Modelling the relationship between microscopy- and RDT-derived *Pf*PR

Individual-level data on infection status were aggregated within ADMIN1 zones across sub-Saharan Africa, and microscopy-derived (*Pf*PR^MIC^) and RDT-derived (*Pf*PR^RDT^) infection prevalence were calculated for each zone. Any estimates from <10 individuals were excluded. A Bayesian probit regression was fit to the resulting 458 pairwise *Pf*PR observations with an errors-in-variables approach [19]. This approach was employed to account for sampling error in both the RDT-derived measurement and the microscopy-derived measurement. In hierarchical Bayesian notation,

1 $$n_{i}^{\text{MIC}} \sim Binom\left( {p_{i}^{\text{MIC}} , n_{i}^{\text{tot}} } \right)$$

2 $$n_{i}^{\text{RDT}} \sim Binom(p_{i}^{\text{RDT}} , n_{i}^{\text{tot}} )$$

3 $$\phi^{ - 1} \left( {p_{i}^{\text{MIC}} } \right) = \alpha + \beta \times \phi^{ - 1} \left( {p_{i}^{\text{RDT}} } \right)$$

4 $$\phi^{ - 1} \left( {p_{i}^{\text{RDT}} } \right) \sim Norm(\mu_{i} ,\sigma_{i} )$$

5 $$\left\{ {\mu_{i} ,\sigma_{i} } \right\}(i = 1, \ldots ,n^{\text{obs}} ) \sim F$$

6 $$F \sim DP(G_{\varTheta } ,m)$$

7 $$\alpha ,\beta ,\varTheta ,m \sim \pi ,$$

where *n*_*i*_^MIC^ and *n*_*i*_^RDT^ are the number of microscopy and RDT positive counts, respectively, with *n*_*i*_^tot^ the total number of individuals tested in each of the *i* = 1 … *n*^obs^ site-specific aggregations; likewise, *p*_*i*_^MIC^ and *p*_*i*_^RDT^ are the long-run *Pf*PR^MIC^ and *Pf*PR^RDT^; $\phi^{-1}(\cdot)$ denotes the inverse probit function that maps the linear predictor within the range of valid probabilities (zero to one); $\alpha$ and $\beta$ are the unknown parameters of the regression model; *μ*_*i*_ and *σ*_*i*_ are the mean and standard deviation of the Normal mixture component from which the *i*-th $\phi^{-1}$(*p*_*i*_^RDT^) is drawn; each *μ*_*i*_ and *σ*_*i*_ pair is drawn from a single realisation, *F*, of the Dirichlet process, *DP*(*G*_*Θ*_, *m*), with reference density, *G*_*Θ*_, (here a single Normal controlled by the hyper-parameters, Θ) and concentration index *m*; while π represents a family of priors and hyperpriors chosen for conjugacy with the upper layers.

The use of Bayesian inference with an errors-in-variables structure requires the specification of a prior for the baseline distribution of long-run RDT-derived prevalence, which in the above is handled by the highly flexible semi-parametric form known as the Dirichlet process mixture model [20]. To facilitate posterior simulation via Gibbs sampling for ordinary probit regressions, Albert and Chib [21] propose an augmented variable method based on the introduction of an additional unit-variance, Normal latent variable per binary response, i.e., a non-observed or inferred variable. Here this approach is extended to the errors-in-variables context with a further latent variable for each binary observation in the RDT-derived measurement and couple the procedure to a Polya urn sampler for the Dirichlet process (from the DPpackage library in R [22]). Leave-one-out cross-validation was employed to evaluate the statistical performance of the fitted model to predict the microscopy-derived prevalence from the RDT-derived prevalence.

### Analysis of factors influencing the relationship between *Pf*PR^MIC^ and *Pf*PR^RDT^

**Fig. 2:**
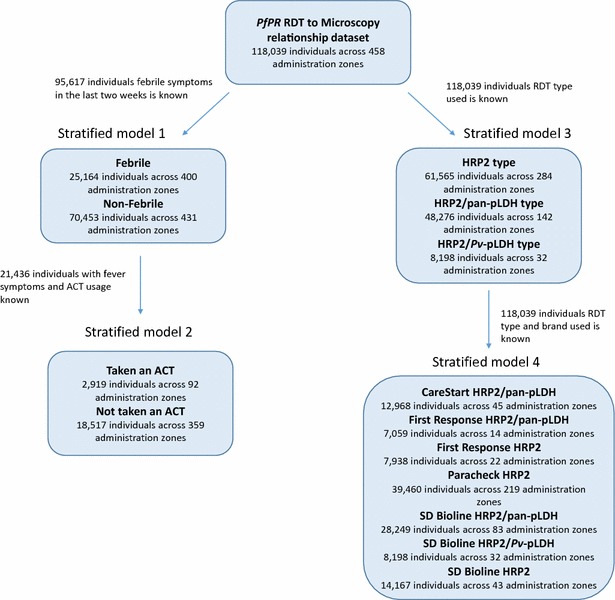
The stratified sub-sets of the data based on fever status, ACT usage, RDT type, and RDT brand

To explore possible factors driving residual noise in the relationship between microscopy and RDT-derived prevalence, the Bayesian regression analysis described above was repeated on various stratified sub-sets of the data: by fever status, treatment status, RDT type, and RDT brand (see Fig. 2 for more details). For each factor pre- and post-stratification akaike information criterion (AIC) scores [23] were compared to determine whether the stratification substantially improved the net information content of the fitted regression model.

## Results

### Modelled *Pf*PR^MIC^*versusPf*PR^RDT^ relationship

**Fig. 3:**
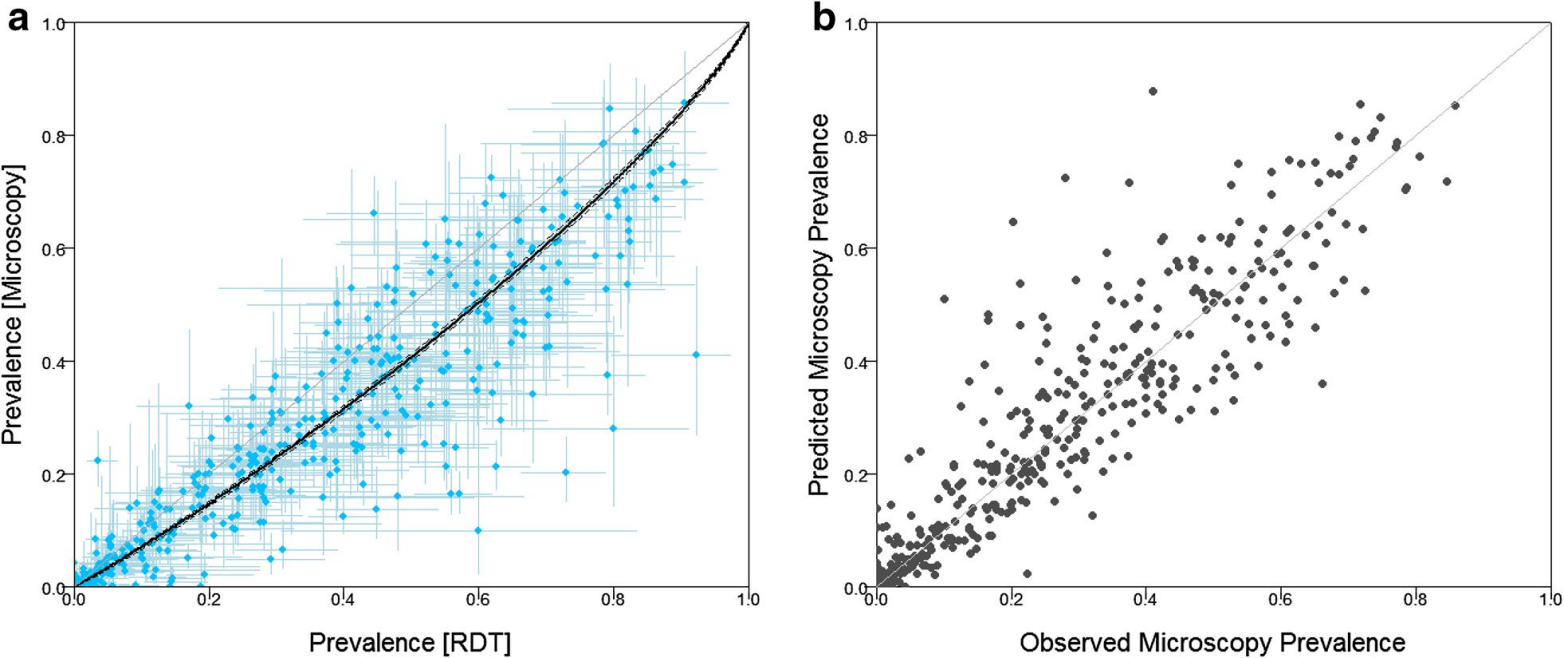
**a** Pairwise *Pf*PR^MIC^ and *Pf*PR^RDT^ observations (*blue dots*) with sampling error bounds (*blue crosses*) with the (point-wise) median curve and 95 % credible intervals from the Bayesian probit regression (*solid black line* and *dashed black lines*) overlaid. **b** The correlation between observed and predicted microscopy prevalence inferred from leave-one-out cross-validation. The *line* of equality (*grey line*) is included for reference in both* panels*

Figure 3a shows the aggregated prevalence data points and their associated uncertainties, the (point-wise) median and 95 % credible interval curves from the posterior of the probit regression model, as well as the line of equality for reference. A systematic tendency for *Pf*PR^RDT^ to exceed *Pf*PR^MIC^ when measured in the same population was observed, indicating that RDTs tend to give more false positives than false negatives, i.e., most (71.6 %) points in Fig. 3a lie below line of equality. The best-fit regression function relating *Pf*PR^RDT^ to *Pf*PR^MIC^ was:

8 $$\phi^{ - 1} \left( {p_{i}^{\text{MIC}} } \right) = - 0.22 + 0.97 \times \phi^{ - 1} \left( {p_{i}^{\text{RDT}} } \right)$$

The results from the leave-one-out cross-validation procedure applied to this model are shown in Fig. 3b. The correlation coefficient between the observed and predicted *Pf*PR^MIC^ was 0.921, the mean square error was 0.84 %, and the mean absolute error was 5.77 % indicating a well-performing model with strong predictive capacity.

### Analysis of factors influencing the relationship between *Pf*PR^MIC^ and *Pf*PR^RDT^

**Fig. 4:**
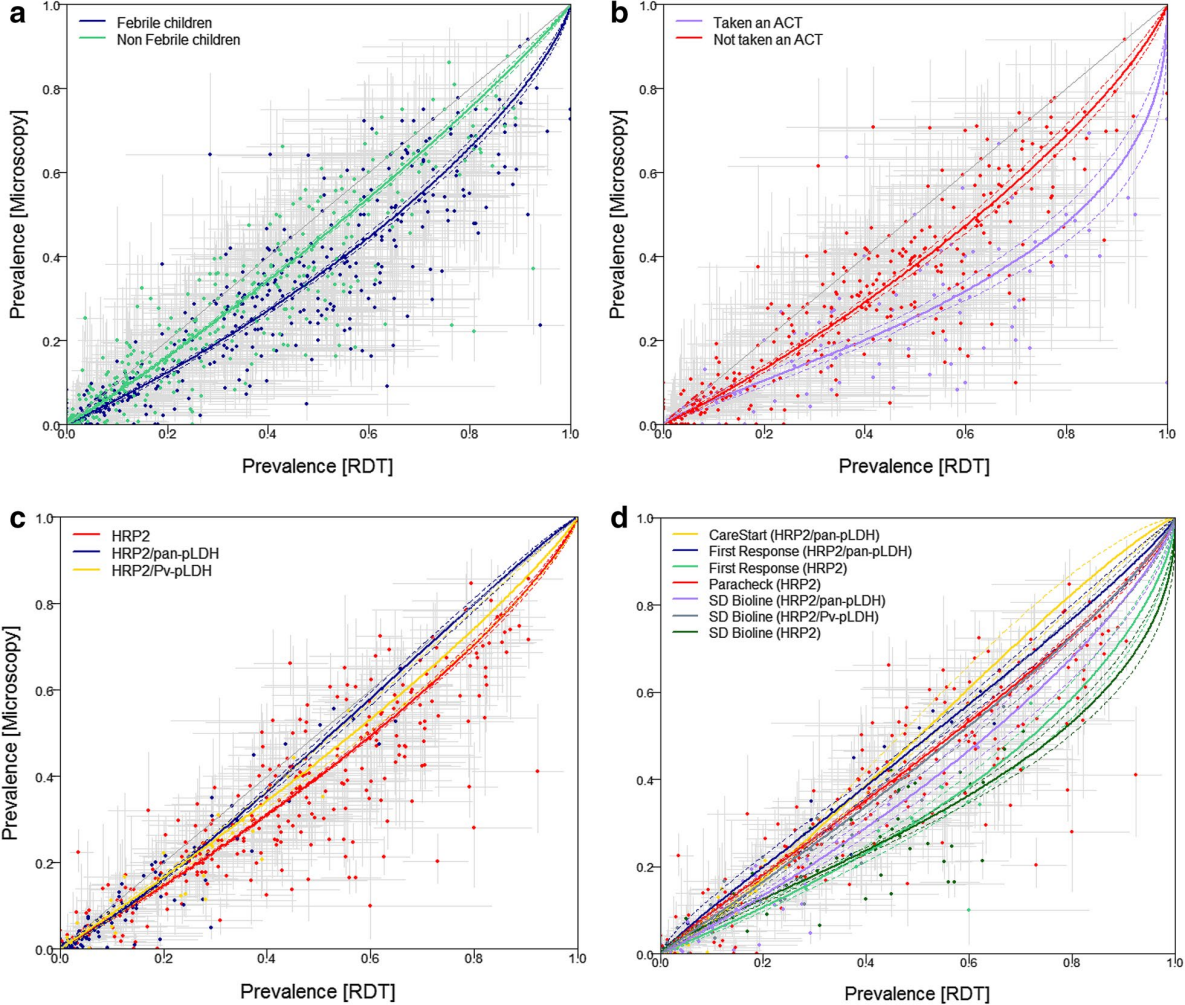
The fitted regression functions with the 95 % credible intervals overlaid on the paired microscopy and RDT derived prevalence data points with their individual error-bounds, for the following stratifications. **a** febrile (*dark blue*) and non-febrile children (*green*), **b** febrile children who have taken an ACT in the last 2 weeks (*purple*) and febrile children who have not taken an ACT in the last 2 weeks (*red*), **c** RDT detected HRP2 (*red*), RDT detected HRP2 and pan-pLDH (*dark blue*) and RDT detected HRP2 and *P. vivax*-pLDH (*yellow*), **d** RDT brand CareStart detected HRP2 and pLDH (*yellow*), RDT brand First Response detected HRP2 and pan-pLDH (*dark blue*), RDT brand First Response detected HRP2 (*green*), RDT brand Paracheck detected HRP2 (*red*), RDT brand SD Bioline detected HRP2 and pan-pLDH (*purple*), RDT brand SD Bioline detected HRP2 and *P. vivax*-pLDH (*dark grey*) and RDT brand SD Bioline detected HRP2 (*dark green*). The *line* of equality is included in each plot for reference

Figure 4a shows the data stratified by individual fever status and the resulting fitted curves. The prevalence rate estimated by RDT amongst non-febrile children is more closely aligned with the prevalence estimated by microscopy than that amongst febrile children. The difference in AIC scores between the original (non-stratified) model and that stratified by fever status confirms that the stratified model performs substantially better (see Table 2).

**Table 2:** The estimated AIC for each of the models

Model	AIC of stratified model	AIC model not stratified	Preferred model
Febrile/non-febrile (stratified model 1)	8523.86	8756.76	Stratified model
Taken ACT/not taken ACT (stratified model 2)	3700.14	3787.54	Stratified model
RDT type (stratified model 3)	6480.68	6299.77	Not stratified
RDT brands (stratified model 4)	5970.06	6299.77	Stratified model
Febrile/non-febrile with RDT brands	8023.65	8756.76	Stratified model

Recent treatment with ACT has a marked effect on the observed relationship between microscopy and RDT-derived prevalence as seen in Fig. 4b, greatly increasing *Pf*PR^RDT^ relative to *Pf*PR^MIC^. The ‘taken an ACT’ sub-set relationship is less precisely determined (note the larger spread and larger confidence intervals in Fig. 4b). In addition to the smaller sample size, this likely reflects the irregular occurrence of false positivity due to perseverance of antigens after anti-malarial treatment and parasite clearance [16, 17]. Although the stratified model is preferred for its lower AIC score (see Table 2), the unpredictability of the false positivity among the taken-ACT children indicates that RDT diagnosis is not ideal and that microscopy would provide a more conclusive diagnosis.

In contrast, there is far less divergence between the prevalence rates estimated by RDTs detecting HRP2 alone compared with HRP2 and pan-pLDH, or HRP2 and *P*. *vivax*-pLDH, as seen in Fig. 4c, and indeed the stratified model in this case is not favoured by the AIC score. Therefore, there is no benefit in stratifying by RDT type.

Figure 4d shows the relationship of the *Pf*PR^MIC^ and *Pf*PR^RDT^ by RDT brand. The CareStart (HRP2/pan-pLDH) has a near one–one relationship (parameter values being estimated near to *α* equal 0 and *β* equal 1) whereas First Response (HRP2) and SD Bioline (HRP2) diverge to *Pf*PR^RDT^, being almost double *Pf*PR^MIC^ at *Pf*PR^RDT^ at 40 %. The AIC score of the RDT brand stratified model is significantly lower than the non-stratified model, favouring stratification.

The combination of febrile status and RDT brand was additionally explored. The AIC score of the febrile status with RDT brand stratified model was lower than the non-stratified model, indicating the stratified model to be preferred (see Table 2). The combination of ACT usage and RDT brands was not possible to explore with our dataset due to low sample sizes for the children taken an ACT by RDT brand.

In summary, the AIC scores are lower for the models stratified by febrile status, ACT usage among febrile individuals, RDT brands and febrile status with RDT brands than the non-stratified model, and are thus considered the preferred models. When applying the conversion formula to an RDT-derived prevalence, cross-sectional population, survey dataset, if the population has a known febrile status, known ACT usage among febrile individuals, or RDT brand used is known, the preferred method for estimating the equivalent microscopy prevalence would, therefore, be applying the model specific to that population. The resulting regression function parameters with 95 % credible intervals for each of the data sub-sets are listed in Table 3.

**Table 3:** The estimated parameters for the fitted regression function (Eq. 3) and their 95 % credible intervals

Dataset	α	β
Overall	−0.22 (−0.24, −0.21)	0.97 (0.95, 0.99)
Febrile	−0.37 (−0.39,−0.34)	0.93 (0.89, 0.97)
Non-febrile	−0.15 (−0.17, −0.13)	0.99 (0.96, 1.01)
Taken an ACT	−0.65 (−0.72, −0.59)	0.71 (0.59, 0.83)
Not taken an ACT	−0.31 (−0.34, −0.27)	0.96 (0.91, 1.00)
HPR2	−0.25 (−0.27, −0.24)	0.95 (0.92, 0.97)
HPR2/pan-pLDH	−0.08 (−0.13, −0.04)	1.07 (1.03, 1.11)
HPR2/*Pv*-pLDH	−0.16 (−0.22, −0.10)	0.96 (0.88, 1.03)
CareStart (HPR2/pan-pLDH)	−0.01 (−0.15, 0.13)	1.12 (1.01, 1.23)
First Response (HPR2/pan-pLDH)	−0.03 (−0.08, 0.01)	0.92 (0.83, 1.02)
First Response (HPR2)	−0.52 (−0.58, −0.46)	0.86 (0.81, 0.93)
Paracheck (HPR2)	−0.15 (−0.16, −0.13)	0.91 (0.88, 0.94)
SD Bioline (HPR2/pan-pLDH)	−0.31 (−0.38, −0.24)	0.94 (0.88, 0.99)
SD Bioline (HPR2/*Pv*-pLDH)	−0.16 (−0.22, −0.10)	0.95 (0.87, 1.03)
SD Bioline (HPR2)	−0.53 (−0.56, −0.49)	0.72 (0.65, 0.79)
Febrile with CareStart (HPR2/pan-pLDH)	−0.31 (−0.54, 0.02)	1.01 (0.77, 1.34)
Febrile with First Response (HPR2/pan-pLDH)	−0.16 (−0.25, −0.07)	0.90 (0.71, 1.12)
Febrile with First Response (HPR2)	−0.58 (−0.66, −0.50)	0.84 (0.70, 0.98)
Febrile with Paracheck (HPR2)	−0.31 (−0.35, −0.27)	0.86 (0.80, 0.91)
Febrile with SD Bioline (HPR2/pan-pLDH)	−0.37 (−0.47, −0.27)	0.96 (0.85, 1.06)
Febrile with SD Bioline (HPR2/*Pv*-pLDH)	−0.32 (−0.43, −0.20)	0.91 (0.75, 1.09)
Febrile with SD Bioline (HPR2)	−0.49 (−0.55, −0.44)	0.81 (0.69, 0.95)
Non-febrile with CareStart (HPR2/pan-pLDH)	0.30 (0.08, 0.51)	1.30 (1.15, 1.45)
Non-febrile with First Response (HPR2/pan-pLDH)	0.02 (−0.04, 0.08)	0.89 (0.79, 1.00)
Non-febrile with First Response (HPR2)	−0.42 (−0.53, −0.31)	0.93 (0.85, 1.03)
Non-febrile with Paracheck (HPR2)	−0.09 (−0.12, −0.07)	0.93 (0.89, 0.97)
Non-febrile with SD Bioline (HPR2/pan-pLDH)	−0.21 (−0.36, −0.07)	0.98 (0.87, 1.07)
Non-febrile with SD Bioline (HPR2/*Pv*-pLDH)	−0.12 (−0.19, −0.05)	0.96 (0.87, 1.07)
Non-febrile with SD Bioline (HPR2)	−0.53 (−0.58, −0.49)	0.65 (0.56, 0.73)

## Discussion

The models developed in this paper provide a means of converting RDT-derived *Pf*PR measurements into estimates compatible with microscopy-derived *Pf*PR or vice versa. As such, the approach provides an indispensable tool for data standardization, designed to decrease the overall uncertainty associated with models that utilize *Pf*PR data derived using differing diagnostic techniques. This research also illustrates the utility of ancillary factors when converting *Pf*PR metrics to reduce the residual noise. Those wishing to apply the conversions developed here can do so using the coefficients in Table 3.

The models were derived using a hierarchical Bayesian framework implementing an errors-in-variables probit regression with a highly flexible, semi-parametric prior (the Dirichlet process mixture model) for marginalizing over uncertainty in the distribution of RDT-derived prevalence. The statistical reliability and predictive performance of this model has been demonstrated through leave-one-out cross-validation.

The field setting of the joint microscopy and RDT prevalence measurements in the DHS dataset distinguishes the present analysis from previous formal verification studies for RDT accuracy [14]; thus the results presented offer a complementary picture of RDT performance outside of control conditions, but require care in their interpretation. For instance, while one may be confident in supposing that the observed strength of recent treatment with ACT as a factor for overdiagnosis by RDT surveillance is likely due to the known lag between asexual parasite clearance and the antigenic-response targeted by RDTs [24], the apparent role of febrile status in RDT overdiagnosis is less easily explained. Previous studies have also observed higher RDT false positives among febrile patients [25, 26]. Uncovering the causal relationship will be important for the accuracy of analyses forecasting likely cost-benefit analyses of different designs for the diagnosis and treatment strategies of proposed mass screen and treat campaigns [27]. In the absence of further data, some insight into the epidemiological processes behind the role of febrile status in RDT positivity rates may be gained from in silico experiments with mechanistic transmission codes if RDT measurement can be modelled immunologically, rather than by proxy through asexual density. Similar caveats apply to the interpretation of the results for the role of RDT brand in shaping the observed relationship.

The model incorporates random sampling noise for both the RDT and microscopy-derived measurements and the systematic noise of the RDT-derived measurements is addressed by exploring the factors that may influence the relationship. However, the systematic noise of the microscopy-derived measurements have not been explored here. There may be factors that are cause systematic noise to the relationship such as the condition of the microscope, microscopist’s training and judgement [11]. The data used here are from a consistent resource that uses a strict microscopy protocol in national facilities [18] and, therefore, these factors are likely to be minimal. Caution should however be applied when applying the presented regression function to microscopy-derived prevalence data from inconsistent sources with large variation in their diagnosing protocol.

There has been reports in some regions, mainly in South America, of *Pf* with absent expression of HRP2 affecting RDT performance [28]. The occurrence of these reports have been marginal in sub-Sahara Africa [29, 30] and, therefore, unlikely to affect the analysis presented here, however caution should be applied when extended this to other geographic regions.

Previous evaluations of RDT diagnoses in field settings at specific sites (covering a limited range of transmission conditions) have identified a strong dependence of RDT specificity on age, such that overdiagnosis is most common amongst young children and much less common amongst adults [31]. The results presented here are based on the DHS Programme data for children only; hence, the fitted relationships cannot be certified for conversion of all age prevalence survey data.

## Conclusions

The research conducted in this paper offers a robust, data-driven approach for converting RDT-derived measurements of *Pf*PR estimates into the more traditional microscopy-based measurements. The predictive accuracy of the conversion approach was high (correlation coefficient >0.9) for the generic, non-stratified model. The conversion technique developed here was a necessary precursor to the latest round of *Pf*PR mapping in Africa by MAP [32] as it provides a means of including several national-level surveys for which only RDT data were collected. This technique is likely to become increasingly important in forthcoming years due to the increased usage of RDTs in *P. falciparum*-endemic countries.

## Abbreviations

*Pf*PR: *Plasmodium falciparum* parasite rate
RDT: Rapid diagnostic test
PCR: Polymerase chain reaction
MAP: Malaria Atlas Project
WHO: World Health Organization
DHS: Demographic and Health Surveys
ADMIN1: Administrative zone
HRP2: Histidine-rich protein 2
pLDH: *Plasmodium* lactate dehydrogenase
ACT: Artemisinin-based combination therapy
*Pf*PR^MIC^: Microscopy-derived *Plasmodium falciparum* parasite rate
*Pf*PR^RDT^: RDT-derived *Plasmodium falciparum* parasite rate
AIC: Akaike information criterion
